# Ocular Clues to Liver Disease: A Strategic Diagnostic Lens

**DOI:** 10.3390/diseases14050152

**Published:** 2026-04-24

**Authors:** Muhammad Dahshan, Hassan Dahshan, Ayhan Basoglu, Huseyin Kadikoy

**Affiliations:** 1 Joan C. Edwards School of Medicine, Marshall University, Huntington, WV 25701, USA; dahshan4@marshall.edu (M.D.);; 2 Independent Researcher, Charleston, WV 25304, USA; 3 Department of Ophthalmology, Charleston Area Medical Center, Charleston, WV 25304, USA

**Keywords:** liver–eye axis, ophthalmology, extrahepatic manifestations, ocular findings, hepatic disease, diagnostic screening

## Abstract

Background/Objectives: Hepatic diseases frequently present with ocular manifestations that aid diagnosis, provide prognostic data, and guide therapy. Despite the clear utility of the liver–eye axis, the literature lacks reviews that categorize these manifestations by etiology. This review evaluates current evidence to identify ocular findings that serve as clinical tools for diagnosis, prognosis, and therapeutic monitoring of hepatic pathologies. Methods: A narrative review was conducted using PubMed and Google Scholar to identify English-language articles addressing ocular manifestations associated with liver disease. The primary search encompassed publications from 2000 to 2025, with inclusion of select foundational works published prior to 2000 when they represented seminal studies establishing diagnostic criteria, pathophysiological mechanisms, or natural history data not superseded by subsequent research. Search terms included combinations of liver, hepatic, hepatitis, cirrhosis, cholestasis, eye, ocular, retina, cornea, sclera, conjunctiva, ophthalmic manifestations, and specific disease names. All study designs were eligible. Society guidelines, systematic reviews, and studies from high-impact journals were prioritized. The final selection comprised 59 references representing the most authoritative sources across the spectrum of hepatic conditions. Results: A spectrum of ocular findings linked to distinct hepatic conditions was identified. Manifestations with established clinicopathologic associations were categorized into congenital and acquired etiologies. Congenital liver pathologies included metabolic disorders (Wilson disease, galactosemia, lysosomal storage disorders) and syndromic/genetic causes (Alagille syndrome, hereditary hemochromatosis). Acquired liver diseases encompassed infectious (hepatitis B/C), drug-induced and iatrogenic (interferon, immune checkpoint inhibitors), nutritional (vitamin A deficiency), neoplastic (metastatic hepatocellular carcinoma), and cirrhotic causes. Conclusions: Specific ocular signs raise clinical suspicion for underlying liver disease and warrant targeted hepatic evaluation. Recognizing these associations facilitates earlier diagnosis and improves outcomes. Systematic screening for these signs is supported in at-risk populations, and prospective validation studies should establish their sensitivity and specificity.

## 1. Introduction

The eye, often described as the window to systemic health, offers vital clues to underlying hepatic dysfunction through a diverse array of clinical manifestations. In clinical practice, ocular manifestations can serve as early, non-invasive indicators of liver disease, transforming routine eye exams into strategic diagnostic checkpoints that may detect hepatic pathology before symptomatic disease manifests [1,2].

This intersection between ophthalmology and hepatology extends beyond anatomy; it reflects interconnected signaling systems where hepatic metabolic derangements, inflammatory cascades, and vascular alterations produce measurable ocular changes [1,3]. The physiological basis for this connection lies in the eye’s high metabolic demand and unique susceptibility to systemic microvascular and metabolic derangements originating from hepatic dysfunction. The retina is one of the most metabolically active tissues in the human body, requiring a constant supply of oxygen and nutrients, making it vulnerable to the systemic oxidative stress and vascular dysregulation seen in chronic liver disease. Furthermore, the liver’s role in synthesizing clotting factors and regulating serum osmolality directly impacts ocular perfusion and fluid dynamics. When hepatic function is compromised, the resultant accumulation of aberrant systemic metabolites (such as copper, bilirubin, and abnormal lipids) and circulating immune complexes can manifest as distinct structural changes in the cornea, lens, and retina, effectively turning the eye into a biomarker for hepatic homeostasis.

In clinical practice, these manifestations are frequently dismissed as isolated ophthalmologic findings. This oversight creates a critical diagnostic gap. Recognizing ocular–hepatic correlations equips physicians to identify subtle signs, screen at-risk individuals effectively, and track therapeutic responses [2,4]. Because the liver serves as the central regulator of systemic metabolism, hepatic failure invariably induces widespread extrahepatic sequelae. Consequently, many liver diseases present with ophthalmic signs that offer non-invasive clues to the underlying dysfunction [1,4].

This review identifies key associations between specific hepatic etiologies and their corresponding ocular findings. We elucidate the underlying pathophysiological mechanisms and define clinical indications for screening. The discussion is organized by etiology, distinguishing between congenital (inherited) liver diseases and acquired conditions to provide a clinically relevant diagnostic framework.

## 2. Materials and Methods

A comprehensive narrative review was conducted using the PubMed (MEDLINE) and Google Scholar electronic databases to identify English-language articles addressing ocular manifestations associated with liver disease. The primary search strategy encompassed publications from January 2000 to December 2025. A secondary manual search of reference lists from retrieved articles was also performed to capture foundational works published prior to 2000, specifically when they represented seminal studies establishing strict diagnostic criteria, core pathophysiological mechanisms, or natural history data that have not been superseded by subsequent research.

The search syntax utilized a combination of Medical Subject Headings (MeSH) and free-text keywords, incorporating Boolean operators (AND, OR) to maximize search sensitivity. Search terms included combinations of the following: “liver,” “hepatic,” “hepatitis,” “cirrhosis,” “cholestasis,” “eye,” “ocular,” “retina,” “cornea,” “sclera,” “conjunctiva,” “ophthalmic manifestations,” and highly specific disease nomenclature (e.g., “Wilson disease,” “Alagille syndrome,” “hepatocellular carcinoma”).

To ensure the clinical applicability and scientific rigor of this review, strict exclusion criteria were applied to the initial pool of search results. Articles were systematically excluded if they focused solely on in vitro data or animal models without direct human clinical correlates. Furthermore, studies were excluded if the ocular findings were described as non-specific complications of general critical illness (e.g., exposure keratopathy in mechanically ventilated intensive care patients) rather than direct sequelae of hepatic pathology. Articles lacking full-text accessibility were also excluded. To address the inherent heterogeneity of the ophthalmic literature, we prioritized studies that utilized standardized, objective ophthalmologic grading systems (such as slit-lamp biomicroscopy grading or optical coherence tomography parameters) over those relying entirely on self-reported patient symptoms.

All study designs were considered eligible for inclusion, encompassing randomized clinical trials, prospective and retrospective observational studies, extensive case series, and expert narrative reviews. International society practice guidelines, systematic reviews, and meta-analyses from high-impact, peer-reviewed journals were heavily prioritized during the synthesis phase. The final selection comprised 59 references, rigorously curated to represent the most authoritative and clinically relevant sources across the broad spectrum of hepatic conditions with documented ocular involvement.

## 3. Results

**Figure 1:**
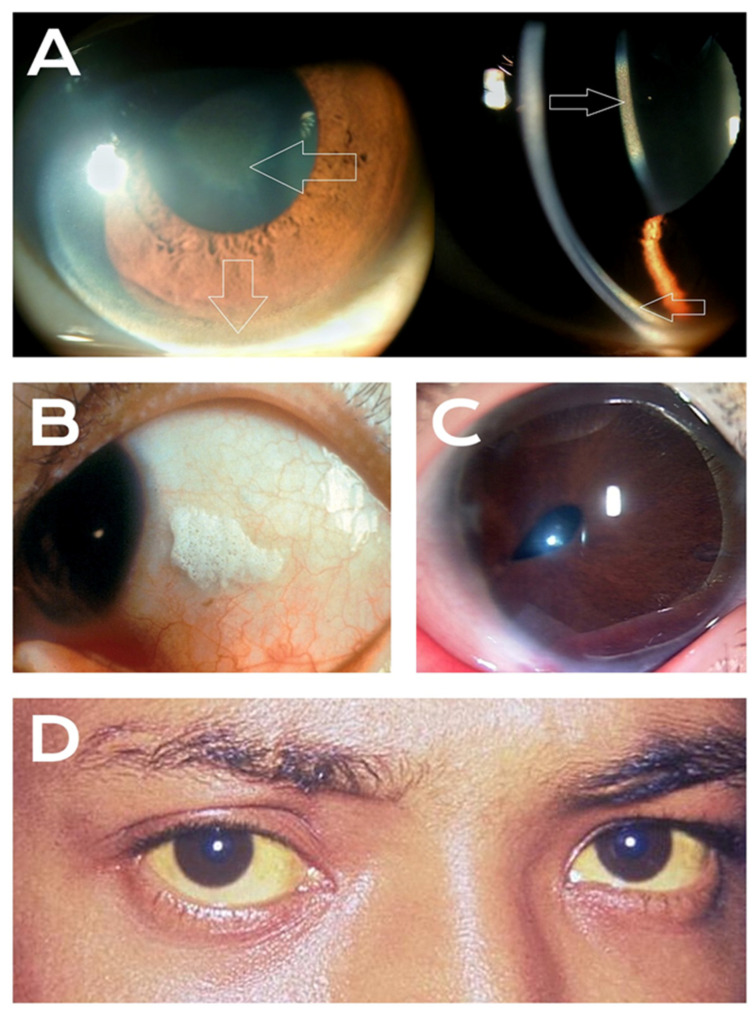
Cardinal Ocular Manifestations of Hepatic Disease. (**A**) Slit-lamp appearance in Wilson disease demonstrating Kayser–Fleischer rings at the corneal periphery and a sunflower cataract in the anterior lens capsule (highlighted by arrows) (Image: Imrankabirhossain, CC BY-SA 4.0, https://commons.wikimedia.org/wiki/File:KF_ring_and_Sunflower_cataract.jpg, accessed on 15 February 2026). (**B**) Foamy, white Bitot spot on the conjunctiva indicative of Vitamin A deficiency (Reprinted from Baiyeroju et al. [5] under CC BY license). (**C**) Anterior segment dysgenesis displaying posterior embryotoxon (anteriorly displaced Schwalbe line) and corectopia (misshapen pupil) (Reproduced from Fuse et al. [6] under CC BY license). (**D**) Severe scleral icterus and jaundice of the facial skin due to hyperbilirubinemia (Image: CDC, Public Domain, https://commons.wikimedia.org/wiki/File:Jaundice_eye_new.jpg, accessed on 15 February 2026).

Our review identified a diverse array of ocular manifestations intrinsically linked to specific hepatic dysfunctions. To establish a strategic diagnostic framework, these clinical findings are categorized based on the etiology of the underlying liver disease, divided broadly into congenital (metabolic and syndromic; Table 1) and acquired conditions (Table 2). A visual summary of the most prominent, cardinal ocular pathologies discussed in this review is presented in Figure 1.

### 3.1. Congenital Liver Disease

#### 3.1.1. Metabolic Causes

**Wilson Disease**: Wilson disease, an autosomal recessive disorder caused by mutations in the ATP7B gene, exemplifies the diagnostic importance of ocular manifestations in metabolic liver disease [7,8]. Defective copper transport leads to accumulation in the liver, brain, and cornea, producing a characteristic spectrum of hepatic, neurologic, and ophthalmologic findings [9].

The hallmark ocular finding is the Kayser–Fleischer (KF) ring, which appears as golden-brown to greenish copper deposits in the Descemet membrane at the corneal periphery [7] (Figure 1A). At the cellular level, the failure of the ATP7B copper-transporting ATPase prevents the excretion of copper into bile and its incorporation into ceruloplasmin. The subsequent accumulation of free unbound copper in the systemic circulation leads to cellular toxicity primarily through the generation of reactive oxygen species via the Fenton and Haber-Weiss reactions. In the eye, this oxidative stress and heavy metal deposition manifest structurally in the Descemet membrane, beginning superiorly and inferiorly before coalescing into a complete ring. KF rings are present in nearly all patients with neurologic Wilson disease and approximately 44–62% of patients with predominantly hepatic presentations [10]. Detection requires slit-lamp examination by an experienced ophthalmologist, as subtle rings are frequently missed on gross inspection [7,10].

In the Leipzig diagnostic scoring system, KF rings carry substantial weight—contributing 2 points [10]. Therefore, if a clinician identifies them in a patient with unexplained liver disease, immediate evaluation is mandatory. KF rings regress with successful copper-chelating therapy, providing a non-invasive marker of treatment response [7].

Sunflower cataracts represent a second ocular manifestation in Wilson disease. These anterior capsular opacities display a characteristic petal-like pattern radiating from the central lens [11]. Unlike KF rings, sunflower cataracts do not typically impair vision. While highly characteristic of Wilson disease, they are not pathognomonic as they can also occur in ocular chalcosis from retained copper-containing intraocular foreign bodies [11].

**Galactosemia**: Classic galactosemia, caused by galactose-1-phosphate uridylyltransferase (GALT) deficiency, produces hepatic dysfunction and cataract formation through the accumulation of galactitol in the lens [12]. Cataracts may develop within the first weeks of life and represent one of the earliest clinical signs, often preceding overt hepatic symptoms [13]. The pathophysiological mechanism driving this rapid cataractogenesis is distinctly osmotic. In the absence of functional GALT, accumulating systemic galactose is shunted into an alternative metabolic pathway where it is reduced by the enzyme aldose reductase into galactitol. Unlike galactose, galactitol is highly membrane-impermeable and becomes trapped within the lens fibers. This creates a severe hyperosmotic intracellular environment, drawing water into the lens, causing swelling, disruption of the highly ordered lens crystallin proteins, and eventual opacification. The characteristic “oil droplet” cataract results directly from this osmotic damage [14]. Newborn screening programs have substantially reduced the incidence of galactosemia-related cataracts through early dietary intervention. However, cataracts may still develop in infants with delayed diagnosis or incomplete dietary restriction [13]. Early cataract formation in a neonate with hepatomegaly, jaundice, or failure to thrive should prompt immediate evaluation for galactosemia.

**Zellweger Spectrum Disorder**: Zellweger spectrum disorder (ZSD) is an autosomal recessive peroxisomal biogenesis disorder caused by PEX gene mutations, resulting in accumulation of very long-chain fatty acids and toxic bile acid intermediates [15]. Hepatic manifestations are prominent and progressive, with histological abnormalities ranging from minor fibrosis to cirrhosis. Liver-related symptoms may be absent even as patients silently develop cirrhosis, necessitating regular surveillance [16]. Ocular manifestations occur independently of hepatic disease severity and include retinal dysfunction and degeneration leading to blindness in almost all patients [15]. Additional findings include corneal opacification, cataracts, glaucoma, pigmentary retinopathy, and optic atrophy [17]. The combination of hepatic dysfunction with these characteristic ocular findings in an infant should prompt evaluation for peroxisomal disorders.

**Niemann–Pick Disease**: Niemann–Pick disease (NPD) comprises a group of rare lysosomal storage disorders with distinct hepatic and ocular manifestations. Type A NPD, caused by severe acid sphingomyelinase deficiency, typically presents with massive hepatosplenomegaly by 3–6 months of age. The hallmark ocular finding is the macular cherry-red spot, present in virtually all patients by 12 months [18]. This distinct finding results from the massive accumulation of sphingomyelin and other complex lipids within the retinal ganglion cells. Because the ganglion cell layer is several cell layers thick in the macula but entirely absent at the central foveola, the lipid-engorged, opacified ganglion cells create a dense white halo. This contrasts sharply with the normal underlying choroidal circulation, which remains visible through the extremely thin foveola, producing the classic “cherry-red” appearance.

Type B NPD presents in mid-childhood with hepatosplenomegaly and potential progression to cirrhosis. Retinal findings include macular halos and cherry-red maculae, though these are less consistently present and often less visually striking than in Type A [18].

Type C NPD, caused by NPC1 or NPC2 mutations, may present with neonatal cholestasis, though many patients present later with progressive, severe neurological manifestations [19]. The characteristic ocular finding is vertical supranuclear gaze palsy (VSGP), manifesting as impaired voluntary downward gaze with preserved reflexive eye movements [20]. The pathophysiology of VSGP involves the toxic accumulation of unesterified cholesterol and sphingolipids within the brainstem, specifically disrupting the rostral interstitial nucleus of the medial longitudinal fasciculus (riMLF)—the primary neural control center for vertical saccades. VSGP typically begins with impaired downward gaze before progressing to involve upward gaze. Because VSGP is present in approximately 70–98% of Type C patients and frequently precedes overt cognitive or motor decline, identifying it serves as a critical, early diagnostic milestone.

**Mucopolysaccharidoses**: The mucopolysaccharidoses (MPS) are a heterogeneous group of lysosomal storage disorders characterized by the progressive accumulation of incompletely degraded glycosaminoglycans (GAGs). While hepatic enlargement (hepatomegaly) is a common presenting feature in MPS types I, II, and VII, ocular findings are nearly universal across the spectrum and often prompt the initial genetic evaluation [21]. Corneal clouding is the hallmark ocular manifestation, particularly in MPS I (Hurler syndrome), VI, and VII, often presenting early in childhood [22]. The pathophysiology of this clouding is strictly structural: the accumulation of hydrophilic GAGs within the corneal stroma disrupts the precise, tightly packed orthogonal arrangement of collagen fibrils that is normally required for corneal transparency. This progressive opacification can lead to severe visual deprivation amblyopia if left untreated. In addition to anterior segment pathology, posterior segment complications such as progressive retinal degeneration and optic nerve atrophy (often secondary to GAG deposition within the sclera physically compressing the optic nerve head, or secondary to hydrocephalus) are also frequent, necessitating regular, comprehensive ophthalmologic monitoring [22].

**Alpha-1 Antitrypsin Deficiency**: Alpha-1 antitrypsin (AAT) deficiency primarily affects the liver and lungs through accumulation of misfolded AAT protein [23]. Ocular manifestations are not well-established. Isolated case reports suggest possible associations with corneal abnormalities and retinal findings, but systematic studies are lacking [24]. While AAT deficiency is not associated with pathognomonic ocular findings, theoretical concerns exist regarding corneal integrity due to the role of protease inhibitors in maintaining corneal stromal homeostasis, which may be relevant for patients considering corneal refractive surgery [24].

**Homocystinuria**: Classic homocystinuria, caused by cystathionine beta-synthase deficiency, produces characteristic skeletal and ocular manifestations [25]. The hallmark ocular finding is ectopia lentis (lens dislocation), which occurs in approximately 55–82% of untreated patients by age 10 [26]. The lens typically dislocates inferonasally, distinguishing homocystinuria from Marfan syndrome, where superior-temporal dislocation predominates. Lens dislocation results from disrupted zonular fibers due to homocysteine-mediated modification (homocysteinylation) of fibrillin-1, which compromises its structural integrity and extracellular matrix deposition [25].

#### 3.1.2. Syndromic and Other Genetic Causes

**Alagille Syndrome**: Alagille syndrome (ALGS) is an autosomal dominant multisystem disorder caused by mutations in JAG1 or NOTCH2, affecting the Notch signaling pathway [27]. Hepatic manifestations include chronic cholestasis due to a paucity of intrahepatic bile ducts. Ocular abnormalities are present in more than 80% of patients and constitute a major diagnostic criterion [27]. The most frequent finding is posterior embryotoxon, a prominent and anteriorly displaced Schwalbe line visible as a white ring at the peripheral cornea. This finding is often accompanied by other anterior segment anomalies like corectopia (a misshapen or off-center pupil) and iris hypoplasia (Figure 1C), though the reported prevalence varies considerably across studies [28,29]. Beyond the anterior segment, optic disc drusen—calcified deposits within the optic nerve head—are frequently observed in ALGS and can mimic papilledema, potentially leading to unnecessary neurological workups if the syndromic association is not recognized. While posterior embryotoxon occurs in 6.8–15% of the general population, its presence in conjunction with other features of ALGS strongly supports the diagnosis. A comprehensive study of ALGS patients found anterior segment abnormalities in 74% of cases and peripheral chorioretinal changes in 96% of patients [28]. The ocular findings reflect underlying developmental abnormalities from disrupted Notch signaling rather than the consequences of cholestasis, distinguishing them mechanistically from vitamin A deficiency-related changes [29].

**Hereditary Hemochromatosis**: Hereditary hemochromatosis, caused by mutations in the HFE gene leading to excessive iron absorption, primarily affects the liver, heart, and endocrine organs. Unlike other metabolic liver diseases discussed in this review, hemochromatosis lacks well-established, clinically significant ocular manifestations [30]. However, emerging research suggests that systemic iron overload may theoretically affect retinal health, though clinical guidelines do not currently list ocular findings as diagnostic features [30]. Screening for HH is driven by hepatic and systemic iron indices rather than ocular signs.

### 3.2. Acquired Liver Disease

#### 3.2.1. Infectious Causes

**Hepatitis B and C Virus Infection**: Chronic hepatitis B virus (HBV) and hepatitis C virus (HCV) infections affect approximately 350 million individuals worldwide (296 million HBV, 58 million HCV) and produce both hepatic and extrahepatic manifestations [31]. Dry eye syndrome represents the most consistently documented ocular manifestation of chronic HCV infection, with impaired tear production in approximately 50% of patients resulting in ocular surface damage [32]. The pathophysiology involves lymphocytic infiltration of lacrimal glands in a pattern resembling Sjögren syndrome, though anti-SSA and anti-SSB antibodies are typically absent [33]. Furthermore, ischemic retinopathy occurs in HCV infection through two distinct mechanisms: direct HCV-induced vasculitis and interferon therapy [34]. The ischemic retinopathy associated with HCV frequently occurs secondary to mixed cryoglobulinemia, a systemic immune-complex mediated vasculitis. Chronic HCV infection drives continuous B-cell proliferation, leading to the production of monoclonal IgM autoantibodies with rheumatoid factor activity that target polyclonal IgG. These large immune complexes (cryoglobulins) precipitate in the cooler microcirculation of the peripheral retina, triggering complement activation, localized endothelial inflammation, and subsequent capillary non-perfusion or frank occlusion. Mooren’s ulcer, a painful peripheral corneal ulceration, has also been associated with HCV infection, though the precise pathogenic mechanism remains to be fully elucidated [32].

#### 3.2.2. Drug-Induced and Iatrogenic Causes

**Interferon-Associated Retinopathy**: Interferon-alpha therapy, though largely historic for hepatitis C, causes a distinct retinopathy in 21–31% of treated patients [34,35]. The classic funduscopic findings include cotton-wool spots and flame-shaped retinal hemorrhages, typically appearing within the first 4–8 weeks of therapy [35]. The pathophysiology involves interferon-induced leukocyte activation and capillary trapping in the retinal microcirculation, leading to focal ischemia [36]. Risk factors include diabetes mellitus, hypertension, age over 45 years, and use of pegylated interferon formulations, and in rare cases, may progress to anterior ischemic optic neuropathy [37]. Retinopathy is typically asymptomatic and resolves spontaneously in approximately 87% of cases after treatment discontinuation [34]. Current recommendations suggest baseline screening for patients with hypertension or diabetes initiating interferon therapy [34].

**Immune Checkpoint Inhibitor-Associated Ocular Toxicity**: Immune checkpoint inhibitors (ICIs), including anti-PD-1 (pembrolizumab, nivolumab) and anti-CTLA-4 (ipilimumab) agents, are increasingly used for hepatocellular carcinoma (HCC). These agents are associated with immune-related adverse events affecting multiple organs, including the eye [38]. Uveitis represents the most common ocular immune-related adverse event, with an overall incidence of approximately 1% [39]. The pathophysiological mechanism involves the drug-induced breakdown of ocular immune privilege. By blocking T-cell inhibitory pathways to enhance anti-tumor responses, ICIs can inadvertently unleash autoreactive T-cells against normal, melanin-rich ocular tissues such as the uveal tract (iris, ciliary body, and choroid). Registry data quantify this risk: anterior uveitis occurs in 2451 per 100,000 patients on pembrolizumab and 8209 per 100,000 on ipilimumab, rates significantly exceeding the baseline population [40]. The median time to onset of uveitis is 5 weeks, with a range from 1 to 72 weeks. Management is guided by severity, with mild anterior uveitis often manageable with topical corticosteroids while continuing immunotherapy [41].

**Tyrosine Kinase Inhibitor Toxicity**: Tyrosine kinase inhibitors (TKIs), including sorafenib and lenvatinib, represent standard first-line systemic therapies for advanced HCC and warrant consideration for ocular monitoring. While ocular adverse events are uncommon compared to those associated with immune checkpoint inhibitors, TKIs have been associated with periorbital edema, dry eye, and rare retinal vascular events including central retinal vein occlusion [42]. A recent pharmacovigilance analysis of over 112,000 adverse drug reaction reports for HCC-targeted TKIs identified ocular events among reported toxicities, though at lower frequencies than dermatologic and gastrointestinal complications [43]. Given the vascular mechanisms of VEGFR-targeting TKIs, patients with pre-existing vascular risk factors may warrant baseline ophthalmologic assessment.

#### 3.2.3. Nutritional Causes

**Vitamin A Deficiency**: Vitamin A deficiency secondary to cholestatic liver disease represents a significant cause of preventable ocular morbidity, particularly in pediatric populations [44]. The liver stores approximately 90% of the body’s vitamin A, and hepatic dysfunction impairs both the storage and the mobilization of this essential nutrient via retinol-binding protein [45]. The pathophysiology of these ocular changes stems from vitamin A’s dual role in phototransduction and epithelial maintenance. In the retina, vitamin A (as 11-cis-retinal) is an obligate chromophore for rhodopsin in rod photoreceptors. Its systemic depletion directly halts the visual cycle, manifesting clinically as nyctalopia (night blindness), which may be the earliest symptom, often preceding clinically apparent structural changes [45]. Concurrently, at the ocular surface, retinoic acid is required for the maintenance of specialized mucosal epithelia. Its deficiency leads to squamous metaplasia of the conjunctiva and a dramatic loss of mucin-producing goblet cells. This loss of tear film stability results in xerophthalmia (conjunctival and corneal dryness) and provides the structural basis for Bitot spots—distinctive triangular patches of keratinized epithelium colonized by *Corynebacterium xerosis* (Figure 1B) [5]. Severe, prolonged deficiency progresses to keratomalacia (corneal melting). In children with chronic cholestasis, these ocular manifestations are common, affecting a substantial proportion of patients. Clinicians should routinely inquire about visual impairment in poorly lit areas in patients with cholestatic disease, as supplementation protocols and monitoring recommendations have been strongly established for these at-risk populations [44].

#### 3.2.4. Neoplastic Causes

**Hepatocellular Carcinoma Orbital Metastases**: Orbital metastases from hepatocellular carcinoma (HCC) represent a rare but diagnostically significant manifestation of advanced disease [46]. While extrahepatic metastases are present in approximately 10–23% of HCC patients at diagnosis, orbital involvement is exceedingly rare [47]. The most common presenting symptom is painful proptosis, reported in approximately half of documented cases [48]. In a review of orbital HCC metastases, 56% of the 9 patients with orbital metastasis first presented to an ophthalmologist with symptoms from the orbital mass before the primary hepatic tumor was identified [48]. Imaging typically reveals a mass in the superolateral orbit with associated bone changes [48]. Diagnosis requires histopathologic confirmation and immunohistochemical staining for hepatocyte-specific markers [48].

#### 3.2.5. Cirrhosis and Portal Hypertension

Cirrhosis, regardless of its underlying etiology, invariably produces systemic hemodynamic alterations that manifest in the ocular microvasculature. Decompensated cirrhosis is characterized by a systemic hyperdynamic circulatory state, driven by splanchnic vasodilation and increased circulating vasodilators such as nitric oxide.

Recent investigations using optical coherence tomography (OCT) and retinal vessel analysis have identified measurable microvascular changes in cirrhotic patients, including significantly altered retinal vessel caliber and reduced overall macular vessel density [49]. Furthermore, the severity of these retinal microvascular changes correlates with renal dysfunction and circulating markers of endothelial dysfunction [49]. Notably, these retinal changes showed substantial resolution following successful liver transplantation, suggesting that OCT parameters may serve not only as diagnostic clues but as dynamic, non-invasive prognostic biomarkers of hepatic function [49].

Intraocular pressure has also been found to be significantly lower in cirrhotic patients, though the precise mechanism remains to be elucidated. Finally, scleral icterus represents the most classically recognized ocular sign of advanced liver disease (Figure 1D). This yellow discoloration of the episcleral and conjunctival tissue becomes clinically apparent when serum bilirubin exceeds approximately 2.5–3.0 mg/dL, and frequently serves as the initial presenting sign prompting urgent hepatic evaluation.

#### 3.2.6. Metabolic and Alcohol-Associated Liver Disease

**Metabolic Dysfunction-Associated Steatotic Liver Disease (MASLD)**: Metabolic dysfunction-associated steatotic liver disease (MASLD), formerly termed nonalcoholic fatty liver disease (NAFLD), affects approximately 38% of the global adult population and represents a multisystem metabolic disorder with emerging ocular associations [50]. MASLD shares pathophysiological mechanisms with diabetic microvascular disease, including insulin resistance, chronic low-grade inflammation, oxidative stress, and endothelial dysfunction [51]. Recent large-scale prospective data from the UK Biobank demonstrate that both MASLD and alcohol-associated liver disease (ALD) are independently associated with increased cataract risk, potentially mediated through elevated circulating homocysteine, reduced glutathione levels, and systemic inflammation affecting lens epithelial cells [52]. Furthermore, MASLD is associated with an increased risk of diabetic retinopathy in patients with prediabetes and type 2 diabetes, with the risk correlating directly to fibrosis severity [53]. Notably, retinal microvascular changes have been shown to predict NAFLD even in obese patients without concurrent hypertension or diabetes [54,55,56]. These findings suggest that the retinal microvasculature may serve as a non-invasive window into the systemic metabolic dysfunction characteristic of MASLD, though dedicated prospective studies examining ocular manifestations specific to this condition remain limited.

**Alcohol-Associated Liver Disease**: Alcohol-associated liver disease (ALD) produces ocular manifestations through both direct toxic effects and secondary nutritional deficiencies. Chronic alcohol use impairs retinal function, with electrophysiological studies demonstrating reduced scotopic electroretinogram amplitudes, indicating rod system dysfunction and inner retinal impairment even before clinical vision loss becomes apparent [57]. Severe anemia complicating ALD may rarely precipitate bilateral nonarteritic anterior ischemic optic neuropathy (NAION), representing a sight-threatening complication that requires urgent hemoglobin monitoring [58]. Additionally, thiamine deficiency in ALD can produce Wernicke encephalopathy with characteristic ophthalmoplegia and nystagmus. Notably, the AASLD practice guidance also explicitly lists icteric conjunctivae as a clinical sign associated with ALD [59].

**Table 1:** Summary of Congenital Liver Diseases and Associated Ocular Manifestations.

Condition & Etiology	Primary Ocular Manifestations	Pathophysiologic Mechanism	Frequency	Reversibility	Diagnostic Utility	Refs.
Wilson Disease (Metabolic)	Kayser–Fleischer ring, Sunflower cataract	Copper deposition in Descemet membrane and lens capsule	KF rings: 95–100% (neurologic), 44–62% (hepatic)	Yes, with chelation therapy	High: KF rings are a major diagnostic criterion (Leipzig score)	[7,10]
Galactosemia (Metabolic)	“Oil droplet” cataracts	Accumulation of galactitol in lens fibers causing osmotic swelling	Variable; reduced by newborn screening	Partial, with early dietary intervention	High: Early sign in neonates; prompts enzyme testing	[12,13,14]
Zellweger Spectrum (Metabolic)	Retinal degeneration, corneal clouding	Peroxisomal biogenesis defect affecting very long-chain fatty acids	Near-universal retinal involvement	No	Moderate: Pattern of findings supports diagnosis in infants	[15,17]
Niemann–Pick Disease (Metabolic)	Type A: Cherry-red spot Type C: Vertical supranuclear gaze palsy (VSGP)	Ganglioside/lipid accumulation in retinal ganglion cells and central nervous system	Type A: ~100% by 12 months Type C: 70–98%	No	High: Specific findings in pediatric hepatosplenomegaly or neonatal cholestasis	[18,19,20]
Mucopolysaccharidoses (Metabolic)	Corneal clouding, optic atrophy	Glycosaminoglycan deposition in corneal stroma	Universal in severe forms	Partial, with enzyme replacement therapy	High: Universal in severe forms; monitors disease progression	[21,22]
Homocystinuria (Metabolic)	Ectopia lentis (lens dislocation)	Homocysteine-mediated modification of fibrillin-1 causing zonular fiber disruption	55–82% by age 10	No	High: Inferonasal dislocation distinguishes it from Marfan syndrome	[25,26]
Alagille Syndrome (Syndromic/Genetic)	Posterior embryotoxon, corectopia, optic disc drusen	Developmental defect in Notch signaling pathway (anterior segment dysgenesis)	Posterior embryotoxon: 78–89%	No (Developmental)	High: Posterior embryotoxon is a major diagnostic criterion	[27,28,29]
Hereditary Hemochromatosis (Syndromic/Genetic)	Retinal pigment epithelium iron deposition (rarely clinical)	Systemic iron overload; deposition in ocular tissues	Rare/Not established (<1%)	Unknown	Low: Not currently used for diagnosis	[30]

Note: KF: Kayser–Fleischer; VSGP: Vertical Supranuclear Gaze Palsy.

**Table 2:** Summary of Acquired Liver Diseases and Associated Ocular Manifestations.

Condition & Etiology	Primary Ocular Manifestations	Pathophysiologic Mechanism	Frequency	Reversibility	Diagnostic Utility	Refs.
Hepatitis C (HCV) (Infectious)	Dry eye syndrome, Ischemic retinopathy	Lymphocytic infiltration of lacrimal glands; Cryoglobulinemia	Dry eye: ~50%	Sicca symptoms improve with DAA therapy; variable for other manifestations	Low/Moderate: Nonspecific but common; prompts screening	[31,32]
Interferon Therapy (Drug-Induced)	Cotton-wool spots, retinal hemorrhages	Immune complex deposition; capillary trapping	21–31%	Yes (~87% resolve after discontinuation)	Moderate: Requires baseline screening in at-risk patients	[34,35]
Immune Checkpoint Inhibitors (Drug-Induced)	Uveitis, Dry eye	Immune-related adverse event (T-cell activation)	Uveitis: ~1%	Yes, with corticosteroids	High: Requires prompt recognition to prevent vision loss	[38,41]
Tyrosine Kinase Inhibitors (Drug-Induced)	Periorbital edema, retinal vascular events	Vascular endothelial growth factor (VEGF) inhibition	Rare (<1%)	Highly variable (varying regimen and risk factors preclude generalization)	Low: Rare but warrants monitoring if symptoms arise	[42,43]
Vitamin A Deficiency (Nutritional)	Night blindness (nyctalopia), Bitot spots	Impaired fat-soluble vitamin absorption due to cholestasis	Up to 60–70% in pediatric cholestasis	Yes, requiring water-miscible or parenteral supplementation	High: Early sign of malnutrition in cholestatic disease	[44,45]
Hepatocellular Carcinoma (Neoplastic)	Orbital metastases (proptosis)	Hematogenous metastatic spread to orbital bone/soft tissue	Rare (<1%)	No	High: May be the initial presentation of occult malignancy	[46,47,48]

Note: ICIs: Immune Checkpoint Inhibitors; TKIs: Tyrosine Kinase Inhibitors; HCV: Hepatitis C Virus; HCC: Hepatocellular Carcinoma. High/Moderate/Low refers to the diagnostic specificity of the ocular sign for the underlying hepatic etiology.

## 4. Discussion

Ocular manifestations of liver disease range from highly specific signs, such as Kayser–Fleischer rings, to nonspecific indicators, including dry eye syndrome. Ultimately, the clinical utility of these findings depends on their specificity, ease of detection, and the availability of confirmatory testing. Key ocular findings provide exceptional diagnostic value; for example, Kayser–Fleischer rings remain the most clinically useful ocular sign in hepatology, and their detection by slit-lamp examination can rapidly expedite diagnosis in patients with unexplained liver disease [7,10]. Similarly, identifying a macular cherry-red spot in Niemann–Pick disease type A or vertical supranuclear gaze palsy in type C represents highly specific findings that can direct immediate diagnostic evaluation in infants with hepatosplenomegaly or cholestasis [18,20].

Beyond congenital presentations, acquired and iatrogenic ocular toxicities represent an increasingly important clinical consideration. Interferon-associated retinopathy remains pertinent for specific indications [32], while the emergence of immune checkpoint inhibitors for hepatocellular carcinoma has introduced new paradigms for ocular monitoring, with uveitis representing the most common immune-related ocular adverse event [39]. Concurrently, advances in retinal imaging offer promising avenues for non-invasive assessment. Optical coherence tomography (OCT) demonstrates distinct utility in detecting microvascular changes associated with cirrhosis, with reduced retinal thickness correlating tightly with renal dysfunction and endothelial markers [49]. Notably, these retinal changes show substantial resolution following liver transplantation, suggesting that OCT parameters may serve as dynamic biomarkers of hepatic function [49].

The pathophysiological mechanisms underlying these liver–eye connections are diverse, encompassing direct metabolic toxicity (copper in Wilson disease), nutritional deficits (vitamin A in cholestasis), immune-mediated processes (cryoglobulinemia in HCV), and developmental abnormalities (Notch pathway in Alagille syndrome) [1]. Understanding these mechanisms enables clinicians to anticipate specific ocular findings. However, the current evidence base presents inherent limitations. Many liver–eye associations are documented primarily through isolated case reports, restricting precise prevalence estimates. The true sensitivity and specificity of most ocular findings for their associated hepatic conditions require rigorous evaluation in larger, prospective cohorts.

The clinical integration of these ophthalmologic findings into routine hepatology workflows represents a significant opportunity to improve patient care. Historically, the liver–eye axis has been viewed as a collection of fragmented clinical observations rather than a cohesive diagnostic framework. Recognizing the eye as a metabolic sentinel organ allows for a more proactive clinical posture. In pediatric populations, an immediate dilated funduscopic exam can dramatically accelerate the diagnosis of lysosomal storage disorders. In adults, the re-emergence of xerophthalmia in chronic cholestasis demands aggressive nutritional screening. As the healthcare landscape shifts toward non-invasive diagnostics, leveraging the transparency of the ocular media offers a highly cost-effective screening modality to triage patients requiring urgent hepatology referral. Looking forward, future longitudinal studies should focus on establishing standardized, quantifiable ocular biomarkers, including specific OCT angiographic parameters and automated retinal vessel caliber ratios, to reliably stage the severity of liver disease and predict the onset of hepatic decompensation. By moving beyond subjective clinical observation and toward these quantifiable thresholds, the medical community can standardize the liver–eye axis as an objective pillar of hepatologic assessment.

## 5. Conclusions

The liver–eye axis offers a clinically robust, non-invasive window into systemic disease. The ocular signs detailed herein validate the ophthalmologic exam as a central component of comprehensive hepatic evaluation. While these ocular manifestations are not universally present, their identification establishes a critical basis for enhanced healthcare delivery.

Due to its high metabolic demand and unique susceptibility to circulating metabolites, the eye often serves as an early indicator of systemic dysfunction. Because the liver is central to metabolic regulation, its failure may therefore be inferred from structural and functional ocular alterations, highlighting the eye’s role as a sentinel organ. This approach is especially valuable in resource-limited settings, where non-invasive physical assessment can rapidly expand diagnostic capabilities.

Systematic screening for selected ocular signs is warranted in at-risk populations. This includes slit-lamp examination for Kayser–Fleischer rings in patients with unexplained liver disease, funduscopic evaluation in children with cholestatic disorders, and baseline ophthalmologic assessment for patients initiating interferon or immune checkpoint inhibitor therapy, as recommended by ASCO and NCCN guidelines for immune-related adverse event monitoring [38].

Prospective validation studies are now required to establish the precise sensitivity, specificity, and clinical utility of these ocular findings as standardized screening tools. Furthermore, emerging technologies, including artificial intelligence-based retinal image analysis and tele-ophthalmology platforms, hold significant promise for the scalable screening of ocular biomarkers in hepatology clinics. Implementing these tools could enable earlier detection of both hepatic and ocular pathology, bridging the gap between ophthalmology and hepatology to ultimately enhance patient outcomes.

## Data Availability

No new data were created or analyzed in this study. Data sharing is not applicable to this article.

## Abbreviations

The following abbreviations are used in this manuscript:

AAT	Alpha-1 Antitrypsin
AASLD	American Association for the Study of Liver Diseases
ALGS	Alagille Syndrome
ATP7B	ATPase Copper Transporting Beta
GAGs	Glycosaminoglycans
GALT	Galactose-1-Phosphate Uridylyltransferase
HBV	Hepatitis B Virus
HCC	Hepatocellular Carcinoma
HCV	Hepatitis C Virus
HFE	Homeostatic Iron Regulator
HH	Hereditary Hemochromatosis
ICI	Immune Checkpoint Inhibitor
JAG1	Jagged Canonical Notch Ligand 1
KF	Kayser–Fleischer
MPS	Mucopolysaccharidoses
NOTCH2	Notch Receptor 2
NPC1/NPC2	Niemann–Pick Disease Type C1/C2
NPD	Niemann–Pick Disease
OCT	Optical Coherence Tomography
PEX	Peroxisomal Biogenesis Factor
TKI	Tyrosine Kinase Inhibitor
VEGF	Vascular Endothelial Growth Factor
VSGP	Vertical Supranuclear Gaze Palsy
ZSD	Zellweger Spectrum Disorder
